# Identifying the Environmental Conditions Favouring West Nile Virus Outbreaks in Europe

**DOI:** 10.1371/journal.pone.0121158

**Published:** 2015-03-24

**Authors:** Matteo Marcantonio, Annapaola Rizzoli, Markus Metz, Roberto Rosà, Giovanni Marini, Elizabeth Chadwick, Markus Neteler

**Affiliations:** 1 Department of Biodiversity and Molecular Ecology, Research and Innovation Centre, Fondazione Edmund Mach, San Michele all’Adige, Italy; 2 School of Bioscience, Cardiff University, Cardiff, Wales, United Kingdom; Université de Perpignan Via Domitia, FRANCE

## Abstract

West Nile Virus (WNV) is a globally important mosquito borne virus, with significant implications for human and animal health. The emergence and spread of new lineages, and increased pathogenicity, is the cause of escalating public health concern. Pinpointing the environmental conditions that favour WNV circulation and transmission to humans is challenging, due both to the complexity of its biological cycle, and the under-diagnosis and reporting of epidemiological data. Here, we used remote sensing and GIS to enable collation of multiple types of environmental data over a continental spatial scale, in order to model annual West Nile Fever (WNF) incidence across Europe and neighbouring countries. Multi-model selection and inference were used to gain a consensus from multiple linear mixed models. Climate and landscape were key predictors of WNF outbreaks (specifically, high precipitation in late winter/early spring, high summer temperatures, summer drought, occurrence of irrigated croplands and highly fragmented forests). Identification of the environmental conditions associated with WNF outbreaks is key to enabling public health bodies to properly focus surveillance and mitigation of West Nile virus impact, but more work needs to be done to enable accurate predictions of WNF risk.

## Introduction

West Nile virus (WNV) is a multi-host mosquito borne virus belonging to the Japanese encephalitis (JE) antigenic complex (genus *Flavivirus*, family *Flaviridae*) [1]. Although the majority (∼80%) of human WNV infections are sub-clinical and can pass unnoticed, some 20% of patients experience flu-like symptoms known as West Nile fever (WNF), while approximately 1% develop a severe, and potentially fatal, neuro-invasive disease [2]. While clinical trials for human vaccines are underway [3] prevention currently depends on organized, sustained vector (mosquito) control campaigns and risk communication [4–8].

Sporadic cases of WNV have been documented in Europe and Africa since it was first identified in Uganda in 1937 [9], but until the 1990s it was considered a low risk for humans and domestic animals. Since then however, WNV has spread rapidly across all populated continents and it is now the most widespread arthropod borne virus in the world [10,11]. In Europe, human cases of WNF have been notified in almost all Eastern, Central, and Southern European countries [12] with hotspots in Italy since 2008 [13], Greece since 2010 [14] and continuous transmission in Russia and Romania since 1996 [15]. The number of WNF cases and the impacts on public health are, so far, limited in Europe relative to other vector borne infection (in 2013, 783 cases of WNF were reported by ECDC in Europe and neighbouring countries, as compared to 45,854 Lyme borreliosis reported by the World Health Organization between 2010 and 2013). However, both escalating case load and increased pathogenicity (e.g. substitution of the NY99 genotype with the more pathogenic WN02 in the USA [16]) are contributing to increased risk. Financial costs associated with the prevention of virus transmission to humans through blood and tissue transplantation are mounting (Blood safety regulation; see [17]).

WNV is maintained in enzootic cycles involving several species of birds, and mosquitoes belonging principally to the *Culex pipiens* complex [7,18–21]. Humans and horses are accidental and dead end hosts since they do not develop a viraemic titre sufficient to infect mosquitoes and amplify the transmission cycle [21]. In Europe, the common house mosquito, *Cx*. *pipiens* (Linnaeus, 1758), is considered the principal bridge vector of WNV between birds and mammals (horses and humans), although at least 60 other mosquito species can be found infected with the virus [7,20,21,22]. *Culex pipiens* occurs in two biological forms, *Cx*. *pipiens pipiens* and *Cx*. *pipiens molestus*, which can hybridize. Both behaviour and host preference vary between forms, with major implications for risk of transmission to humans depending on their relative abundance [23,24]. WNV ecology in the Old World is complex and several aspects of the WNV transmission cycle are as yet poorly quantified. The co-circulation of at least five lineages with variable pathogenicity and the overlap of new introductions with endemic circulation, render the quantification of the parameters necessary to develop transmission models challenging [10,25,26].

Although favourable environmental conditions for virus transmission seem to occur extensively in the Old World and a widespread circulation of the virus has been demonstrated by serological screening of wildlife and sentinel animals, clinical emergence in humans tends to be unpredictable, sporadic and clustered [12]. The occurrence of spatially and temporally localised hot-spots in emergence is likely to reflect the coincidence of circulating virus strain with favourable environmental (biotic and abiotic) conditions which modulate the interaction between virus, mosquito, and hosts, consequently leading to locally altered pathogen amplification, transmission and disease risks [27,28]. Variation in land use, climate, habitat structure, animal community, human socio-economic status or behaviour can all significantly affect the risk of infection—for example, via impacts on the spatial and temporal distribution of the competent reservoir host assemblages and their immune status, as well as on the local abundance and genetic population structure of mosquito vectors and their vectorial capacity [29–33].

At the same time, the availability of simultaneous information on the infection pattern in vectors and birds is lacking at a wide spatial scale, while the cost of integrated surveillance, and the economic and social disparities which affect several EU countries, limit the capacity of high-level institutions to collect detailed and standardised ecological and epidemiological data [35]. Identification of the areas of potential emergence, and predicting temporal and spatial variation in WNV risk therefore remains challenging [34].

The current study aimed to identify environmental factors associated with WNF occurrence in humans across the Old World. We analysed the association between WNF incidence (derived from WNF number of cases as reported annually by the European Centre for Disease Prevention and Control, ECDC) and a wide range of potential predictors including climate, land use, indices of water, vegetation, conservation status, landscape fragmentation and human population density. By identifying key environmental drivers of WNF, we aim to lay the foundations for the development of statistical models able to predict WNF risk at a continental scale.

## Methods

### Epidemiological data

**Fig 1:**
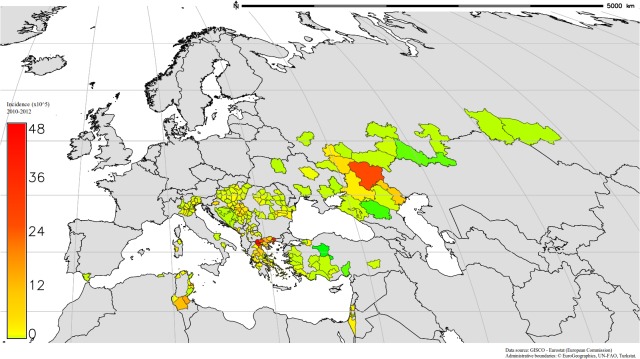
Spatial variation in cumulative WNF incidence (cases per100,000 population) between 2010 and 2012 is indicated in colour, within areas delineated using NUTS3/GAUL1 administrative boundaries. Areas with no reported cases of WNF are shown in grey, and delineated using Country boundaries. Peak incidences are reported in red, these being in Volgograd Oblast, North Eastern Greece and Central Tunisia.

Data used were provided by the European Centre for Disease Prevention and Control (ECDC), [36], compiled from weekly WNF case reports from 146 areas defined at the Nomenclature of Territorial Units level 3 (NUTS3)/Global Administrative Unit Layers level 1 (GAUL1), originating from 16 different countries across western Asia, Europe and northern Africa (Fig. 1). For nation specific details of data collection see [19] and references therein. Weekly data were pooled, to provide annual totals for 2010 to 2012. Population data were obtained for each area using online national statistical databases (Table 1), so that the number of cases per 100,000 inhabitants (hereafter referred to as the 'incidence') of WNF could be calculated per head of population, per year. Areas with no reports of WNF were excluded from analysis, as it was not possible to discriminate between true negatives, and areas where reporting or diagnosis were inadequate.

**Table 1:** Population data.

Country	Source	Web link
Albania	INSTAT	http://www.instat.gov.al
Algeria	Office National des Statistiques	http://www.ons.dz/
Israel	Central Bureau of Statistics	http://www.cbs.gov.il/
Kosovo	Kosovo Agency of Statistics	http://esk.rks-gov.net
Macedonia	State Statistical Office	http://www.stat.gov.mk/
Palestine	Palestinian Central Bureau of Statistics	http://www.stat.gov.mk/
Russia	Palestinian Central Bureau of Statistics	http://www.pcbs.gov.ps
Serbia	Statistical Office of the Republic of Serbia	http://www.gks.ru/
Tunisia	National Institute of Statistics	http://www.ins.nat.tn
Ukraine	State Statistics Service of Ukraine	http://www.ukrstat.gov.ua/
All other countries	Eurostat	http://epp.eurostat.ec.europa.eu

Population data source per country is reported. The population data has been used to derive yearly WNF incidence per each NUTS3 area.

* All population data were from 2009 except Algeria (2008), Kosovo (2011), Russia (2010) and Ukraine (2010).

### Climatic and environmental variables

All climatic and environmental variables were collated as either vector data (protected areas and water bodies) or gridded raster data (all other variables) for the entire study area, and processed using GRASS GIS [37]. Full details of data sources, resolution, and all variables included in statistical analyses are reported in Table 2.

**Table 2:** Climatic and environmental variables.

Variable	Raw data source & resolution	Derived data	Into preliminary model	Into final model	Terms in set of best models
**Temperature**	Gap-filled daily MODIS Land Surface Temperature from MODIS satellite sensor products MOD11A1 and MYD11A1; 4 records per day aggregated to weekly average at 250m pixel resolution [95].	16 week aggregated average and standardised anomaly, calculated individually for nine periods: from weeks 1–16, 2–17, etc to weeks 9–24 in each year and area.	All variables for both Anomalies and Average across all 9 periods.	Ano.Temp3–6; Av.Temp6–9	Av.Temp6–9
**Vegetation index**	Gap-filled Normalized Difference Vegetation Index (NDVI, MODIS product MOD13Q1), at a pixel resolution of 500m.			Ano.NDVI4–7	No
**Water index**	Gap-filled (after [96]) Normalized Difference Water Index (NDWI, calculated from MODIS product MOD09A1), at a pixel resolution of 500m.			Av.NDWI4–7	Yes
**Precipitation**	Gridded ECA&D database, at 25 km resolution [97].	16 week aggregated total precipitation, and days of rain; cumulative total and standardised anomaly for both measures, calculated individually for nine periods: from weeks 1–16, 2–17, etc to weeks 9–24 in each year and area.	All variables for both Anomalies and Cumulative, across all 9 periods—for both total precipitation and days of precipitation.	Av.PrecDays2–5	Yes
Land use*	Land cover classes from GlobCover [38], 300m pixel resolution.	Percentage of each land use category, calculated within each area.	Irrigated Croplands, Rainfed Croplands, Mosaic Croplands, Mosaic Vegetation, Closed Forests/Vegetation, Open Forests/Vegetation, Mosaic Forest, Mosaic Grasslands, Flooded Broadleaved Forests, Artificial surfaces, Water Bodies	Irrigated Croplands; Mixed Natural Vegetation	Irrigated Croplands; Mixed Natural Vegetation#
		Pielou’s index of heterogeneity.	Yes	Yes	Yes #
		Number of land use patches.	Yes	Yes	No
	Anthromes dataset (Anthropogenic Biomes: global ecological patterns created by sustained direct human interactions with ecosystems) [39], 86km pixel resolution).	Percentage of each land use category, calculated within each area.	Urban, Dense settlement, Irrigated villages, Cropped pastoral villages, Rainfed villages, Rainfed mosaic villages, Residential irrigated cropland, Residential rainfed villages, Populated irrigated cropland, Populated rainfed cropland, Residential rangelands, Populated rangelands, Populated forests	Populated Forests; Populated Rangelands	Populated Forests
**Protected areas**	IUCN and UNEP, 2013; http://www.wdpa.org/.	Percentage within each area.	No	Yes	Yes #
**Water bodies**	OpenStreetMap contributors 2013.	m2 / Hectares	No	Yes	Yes #
**Light at night**	Derived from The visible Infra-red Imaging Radiometer Suite (VIIRS) sensor aboard the Suomi National Polar-Orbiting Partnership (NPP).A first set of cloud free DNB data (observations from 2012/4/18–26 and 2012/10/11–23) acquired by VIIRS was released by NOAA for the year 2012. The VIIRS product is cloud free at 15 arc-seconds spatial resolution and corrected for erroneous light sources (http://mapserver.ngdc.noaa.gov/viirs_data/viirs_composite/).	Mean and variance, within each area.	No	Yes	No
**Year**	Years 2010, 2011, 2012.		Yes (as random variable)	Yes (as random variable)	Yes
**Area**	146 areas defined at the NUTS3/GAUL1 level, from 16 different countries across western Asia, Europe and northern Africa.		Yes (as random variable)	Yes (as random variable)	Yes

Data sources and resolution are described; inclusion of each term in preliminary and full models is indicated.

*classes selected based on published evidence of their strong interactions with human incidence of vector-borne disease [58–61,66,71,98–100], excluding those absent from the study area, or represented in less than 10 areas.

# evidence weight < 0.8.

### Raw data

Climatic variables were: i) *Land Surface Temperature* and ii) *Precipitation* (daily amount of precipitation, and number of days with precipitation).

Environmental variables were: i) *Vegetation index*, ii) *Water index*, iii) *Land use* (land cover classes from GlobCover [38] and ‘Anthropogenic Biomes’ (global ecological patterns created by sustained direct human interactions with ecosystems) from the Anthromes dataset [39], iv) *Protected areas*, v) *Water bodies*, and vi) *Intensity of light at night* (used as a proxy for human population density, [40–42]).

### Derived data

All variables were summarized within the NUTS3/GAUL1 areas, from which we derived a series of measures described below.

**Seasonal averages and anomalies.** For datasets incorporating temporal as well as spatial variation (temperature, precipitation, NDVI, NDWI), data from each year and area were aggregated into nine 16 week periods, these being labelled using months instead of weeks, such as 1–4 (January-April), 2–5 (February-May), 3–6 (March-June),…, to 9–12 (September-December) [43]. For each period, we calculated the average (for temperature, NDVI and NDWI) or the cumulative total (precipitation). From these average or cumulative totals we derived anomalies [19] as the difference between the average (or cumulative) value within the given time period, and the average (or cumulative) value for the same time period recorded in the preceding decade, 2001–2010. We then standardized the anomalies by dividing them by the 2001–2010 standard deviation. Standardized anomalies provide more information about the magnitude of the anomalies because influences of dispersion have been removed. This was repeated for each year (2010–2012). Where referred to in the text, each variable (Temp, PrecTot, PrecDays, NDVI, NDWI) is prefixed with Ano. or Av. for standardised anomaly or average, and the relevant period denoted in subscript (e.g. Ano.Temp_2–5_ = weekly anomaly temperature during months 2–5).

**Landscape.** For each land use class (GlobCover and Anthromes), protected areas, and water bodies, percentage cover was calculated within each NUTS3/GAUL1 area. Pielou’s evenness index of diversity [44], and the number of patches of homogeneous land use, were calculated using GlobCover data. For night light intensities, the mean and variance were calculated within each NUTS3/GAUL1 area, and are used as proxies for the average population density, and the fragmentation of the human population, respectively.

### Statistical analyses

We investigated the association between incidence of WNF and a range of environmental predictors, measured across Europe and adjacent countries, using available epidemiological data from 2010–2012. For all analyses, we used as the response variable the log-transformed annual incidence of WNF, for each NUTS3/GAUL1 area, respectively. All analyses were performed using the 'R' language and environment for statistical computing [45].

### Preliminary analyses

In order to select the most appropriate time window for each temporally variable predictor, linear mixed-effects models (LMMs) were fitted to the response variable, for each time window in turn, for both the average and the standardised anomaly of each of the temporally variable environmental predictors. Year and area (NUTS3/GAUL1) were included as random variables. The inclusion of random factors in the model is needed to take under control the clustering of data in different areas and years [46]. The best model was selected for each predictor using An Information Criterion (AICc), with small sample bias adjustment [47].

LMMs were fitted to test the association between WNF incidence and land use. Two models were created, testing in turn for associations with land cover (GlobCover data), and anthropogenic biomes (Anthromes data). Using a process of multi-model inference [47–49], we compared all possible models using the R package ‘MuMIn’ [50]. The best models were selected using a threshold of ΔAICc <2 [47].

### The full model

Following preliminary stages of variable selection, we developed further LMMs including all remaining environmental variables (Table 2), with year and area included as random variables. As previously, all possible models were compared using multi-model selection. The consensus set of best models were selected using a threshold of ΔAICc <2 [47], and differences in AICc (ΔAICc) between consecutively ranked models were used to calculate weights and relative evidence ratios for each variable. All variables included in the best models were ranked according to their importance, and the relative evidence weight for each model term was calculated (this being the sum of the IC weights of those models in which the term is included, [49]). These data were used to calculate the model-averaged estimates of the coefficients and their standard error. Although we acknowledge that utilising a process of multi-model selection and inference can lead to the testing of spurious models (that we tried to filter out pre-selecting the variables used in the full model), it is an extremely powerful approach that enables us to present a consensus of landscape and weather predictors from multiple models, rather than only a single ‘best’ model, while also considering model selection uncertainty [47]. After testing the averaged model for multicollinearity using the Variance Inflation Factor (VIF) [51], we compared WNF incidence fitted values versus WNF incidence observed values to assess the averaged model goodness of fit.

In order to clarify effect size, predictions were made from the best models for each significant predictor variable in turn. All variables but one were fixed at their median values, and predictions made across the full range of the selected variable. For example, to test the association between temperature and WNF incidence, in a model where temperature, precipitation and NDWI were all significant predictors, precipitation and NDWI were added to the model as constants (fixed at their median measured value) while values for temperature were allowed to vary within their observed range.

## Results

### WNF incidence

**Fig 2:**
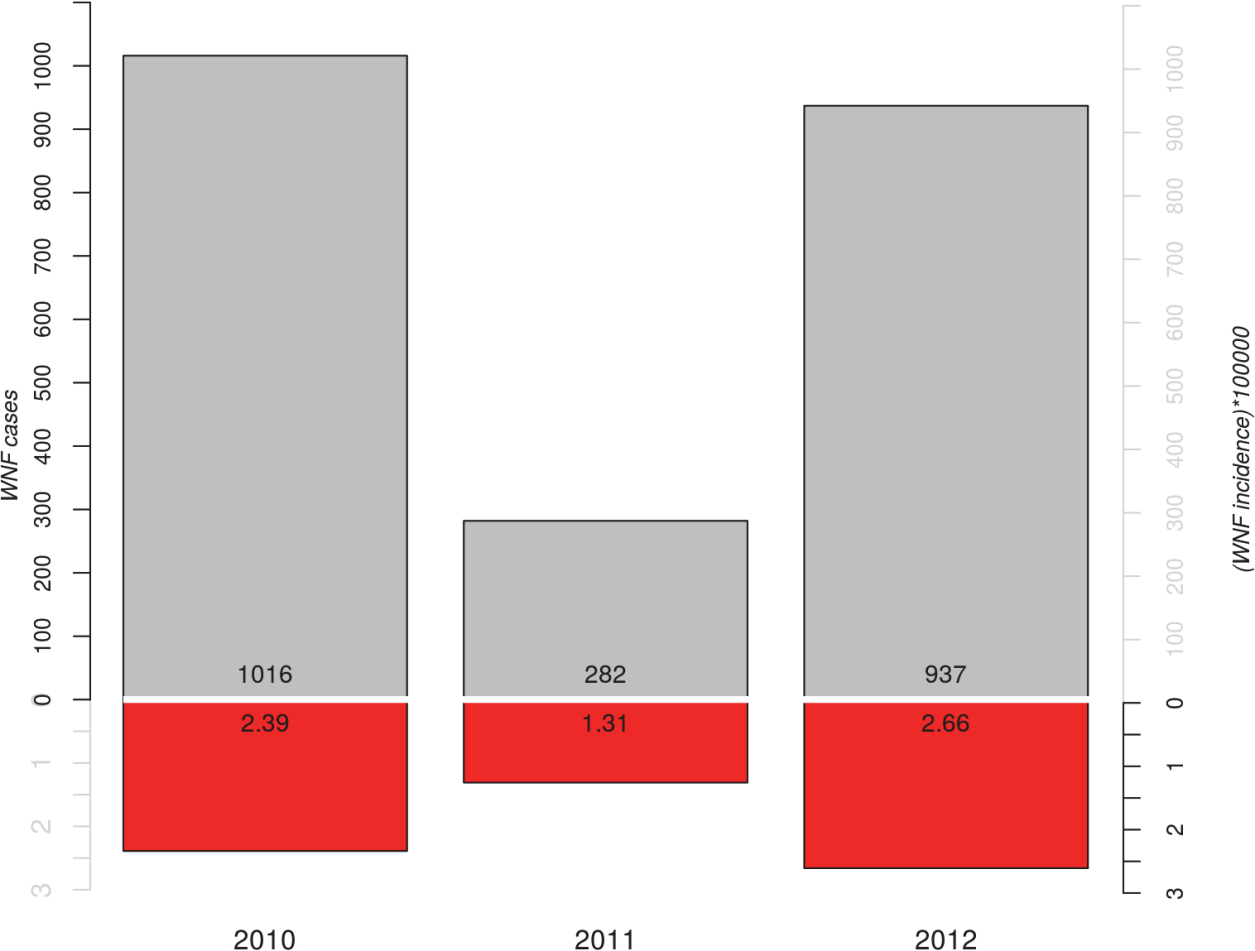
Barplot reporting the total number of cases (grey) and incidence (cases per 100,000 population, red) of WNF in each year (2010, 2011, 2012).

The highest WNF incidences during the 2010–2012 period were reported in Volgograd Oblast, North Eastern Greece and Central Tunisia (Fig. 1). Annual incidence across the entire study area varied from 1.31 cases per 100,000 people in 2011 to 2.66 in 2012 (incidence in 2010 reached an intermediate value of 1.68; Fig. 2). In 2010, the highest incidences were reported in eastern Europe, western Asia (Volgograd Oblast) and Greece. In 2011, the highest incidence was reported in Greece, but overall, incidence was low. In 2012 the average incidence was the highest in Greece and Tunisia.

### Preliminary analyses

To predict incidence of WNF, the optimum periods over which to measure temperature, precipitation, NDWI and NDVI were late-winter early spring (Av.PrecDays_2–5_ and Ano.Temp_3–6_) and summer (Av.NDWI_4–7_, Ano.NDVI_4–7_, Av.Temp_6–9_), respectively. The anthropogenic biomes selected from the initial subset of 14 classes were “Populated Forests” and “Populated Rangelands” (forests or rangelands with scattered, low density human populations), and the land use classes selected from the initial subset of 13 classes were “Mosaic forests” (forests alternated with other land uses such as grasslands), “Irrigated Croplands” and “Mixed Natural Vegetation”.

### The full model

**Fig 3:**
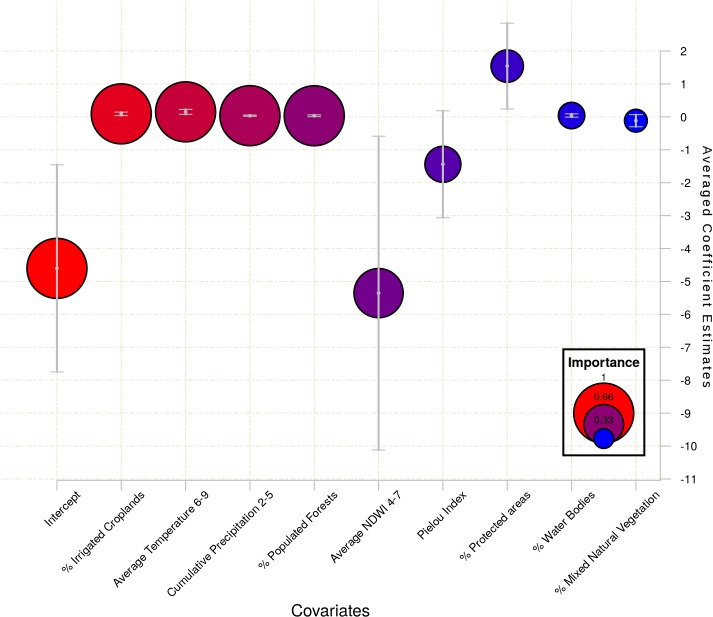
Summary statistics from the best 9 models (ΔAIC≤2 from the best model) for log-transformed WNF incidence (from 2010 to 2012). The coefficients have been derived using multi-model averaging. The model term ‘Importance’ is proportional to the number of times that the variable is included in the set of best models and is represented by the colour and size of the bubbles (red/bigger bubble = high importance; blue/smaller bubble = low importance). Where referred to in the figure, each variable (Temp, PrecTot, PrecDays, NDVI, NDWI) is prefixed with Ano. or Av. for standardised anomaly or average, and the relevant period denoted in subscript (e.g. Ano.Temp_2–5_ = weekly anomaly temperature during months 2–5).

A total of 9 models were identified with ΔAICc <2,. Of the 14 explanatory variables considered in the full model, 8 were represented in the consensus set of best models. The Variance Inflation Factor of the full model was less than 2 for all variables, indicating that multicollinearity was not significant. The model-averaged fitted values explained 32% (R^2^ 0.32) of the observed data, moreover the model averaged residuals did not show any evident pattern, being normally distributed around 0. Model-averaged importance of terms and the estimation of their coefficients (Fig. 3; Table 3) showed that the most important predictors affecting WNF incidence were climatic and land use factors.

**Table 3:** Terms selected in the nine best models.

Model term	Averaged coefficient	Unconditional variance	Relative evidence weight
Intercept	−4.71	1.26	1.00
% Populated Forests	0.03	0.01	1.00
% Irrigated Croplands	0.10	0.03	1.00
Av.Temp6–9	0.15	0.04	1.00
Av.PrecDays2–5	0.04	0.01	1.00
Av.NDWI4–7	−5.35	3.32	0.82
*Pielou Index*	*−1.44*	*0.83*	*0.60*
*% Protected areas*	*1.54*	*0.66*	*0.54*
*% Water Bodies*	*0.04*	*0.03*	*0.44*
*% Mixed Natural Vegetation*	*−0.12*	*0.09*	*0.38*

Model-averaged coefficient estimates for log-transformed WNF incidence (from 2010 to 2012), unconditional variance and the evidence weight (the sum of Akaike weights for that variable) are presented for the best 9 models. Note that terms in italics have an evidence weight < 0.8 and are not deemed important. Where referred to in the table, each variable (Temp, PrecTot, PrecDays, NDVI, NDWI) is prefixed with Ano. or Av. for standardised anomaly or average, and the relevant period denoted in subscript (e.g. Ano.Temp_2–5_ = weekly anomaly temperature during months 2–5).

Water index (NDWI) proved a highly significant predictor, negatively correlated with incidence (cases per 100,000 inhabitants) of WNF such that a decrease in spring-early summer vegetation index (Av.NDWI_4–7_) of 0.10 predicts an increase in incidence of approximately 47 (NDWI theoretically ranges from-1 to 1 but in our study area/years Av.NDWI_4–7_ ranges between-0.13 and +0.04). Summer average temperatures Av.Temp_6–9_) were positively correlated with WNF, such that an increase of 1.6°C (as predicted under climate change scenario RCP 2.6; [52] predicts an increase in incidence of 2. Days of precipitation in late winter/early spring (CumulativePrecDays_2–5_) were also positively correlated with WNF incidence, such that an additional 10 days of precipitation (in the four months considered) predict an increase in incidence of WNF of 29 (where the range within our dataset was between 0 and 58 days of rain in total for this period).

Of the land use variables, the most significant predictors were the percentage of area covered by irrigated croplands, or by populated forests, both of which were positively correlated with incidence of WNF. An increase of 5% in the area cultivated as irrigated croplands predicts an increase in incidence of 113 (in the current study, the average area cultivated in this way varied between 0 and 26% of each NUTS3/GAUL1 area), while an increase of 5% in the area of populated forests predicts an increase of 36 per 100,000 people (in our study, the average area of populated forest varied between 0 and 47%).

Remaining variables were given an evidence weight <0.8, and were not considered significant [53].

## Discussion

West Nile virus is spreading in Europe and neighbouring countries at an increasing rate, with new lineages and variants emerging into new territories. Several factors contribute to the current epidemiological picture, amongst which urbanisation, variation in land use and climate are considered among the most important [19;54–57]. To our knowledge the current study is the first attempt to model WNF human incidence at a continental scale in the Old World, including the whole of Europe, northern Africa and western Asia. Despite the large number of studies set in North America, differences in vectors and reservoir host species, in the degree of human exposure and wildlife immune status, suggest that the ecological processes driving WNV ecology in the New World (e.g. [8]) can be only partly considered to apply to the Old World system. In this system, perhaps because of the far longer history of WNV circulation and the recognition of at least five co-circulating lineages with variable pathogenicity, WNV epidemiology and ecology seem to be more complex.

It is well established that climate affects many components of the WNV biological cycle (e.g. [57–58]), and that the carrying capacity for both vector and host populations differs with land use [60]. The interplay between biotic and abiotic variables that drive WNV in humans forms a complex and dynamic ecological system, and to adequately describe those dynamics across a large scale, requires a comprehensive set of predictors within a modelling framework. The current study makes use of remote sensing and GIS to enable collation of multiple types of environmental data over a continental spatial scale, at sufficient temporal and spatial resolution to test associations with WNF incidence. The number of studies utilising such tools are increasing [60–62] but although a plethora of studies on WNV have been published since the New York outbreak of 1999, only a few authors applied statistical modelling techniques to study spillover potential (i.e. [19,60–66]). Only a small subset of these studies use human cases as the response variable and environmental factors as predictors, and most are conducted at a local or regional spatial scale [60,61,64,66,67,68]. Only two studies [19,70] have made an attempt to model WNV outbreaks in humans in the Old World at a regional scale while others modelled WNV circulation in horses [71].

Climatic anomalies are considered among the most important drivers of WNV emergence, and ambient temperature can be a determinant of outbreaks [33]. Permissive meteorological conditions are necessary for the persistence of active transmission [19], and empirical studies suggest that temperature is a key factor influencing WNV evolution and dissemination [72]. Indeed, the relationship between temperature and increased WNV incidence in humans has repeatedly been confirmed both at national and multinational scales [70,73–74]. In the current study, by using spatially continuous input data, we managed to avoid weak interpolation methods which only employ sparse point data. We found that average summer temperatures between the months of June and September (Av.Temp_6–9_) are positively correlated with WNF human incidence, in concurrence with [19] who found that the summer temperature anomaly before the upsurge was the main driver. Similarly, [70] found a significant relationship between summer temperature anomalies and WNV risk in Europe. The effect of temperature is likely to be mediated through its impact on the distribution, behaviour and survival of the mosquito vector, via direct impacts on the virus and on its’ extrinsic incubation period in the competent vectors (which is reduced at higher temperatures [72]), and via impacts on host distribution and behaviour. The nature of the association with temperature is therefore complex—for example, although warmer temperatures may have a negative effect on mosquito survival, they increase mosquito biting and development rates, and pathogen replication [72] and can lead to increased human exposure risk through altered human activity patterns [8].

Previously, [19] found no significant correlation between WNF incidence and either precipitation or humidity in Europe. In the present study, however, we observe positive associations between WNF incidence and the total number of days with precipitation in late winter-spring (Av.Rain_2–5_), and the percentage of irrigated croplands, suggesting a strong link to spring precipitation, and the presence of standing water. Standing water is a prerequisite for larval development of the mosquito vector, without which they cannot complete their biological cycle [75,76]. WNV transmission to humans is inefficient and infrequent [8], and cases of WNF occurs more often when mosquito population density rises (i.e. see [29]). While [19] found no link to precipitation, their data were limited to 2010, and landscape structure was not considered [77]. Furthermore, the authors used point source climatic data (meteorological stations) which may not represent spatial and temporal variations as well as global earth observations [60]. We believe the results of the current study are a more robust representation of the association between human incidence of WNF and spring precipitation/standing water in Europe, but recognise also that the nature of this association is likely to vary across the geographic range of the virus and the precipitation regimes, as reflected by conflicting evidence in literature (e.g. a positive correlation between precipitation and human incidence in eastern US, but a negative correlation in the west [63], in southern Florida [78] and Israel [79]).

Although precipitation in spring is positively associated with WNF incidence, a strong negative association is seen between WNF incidence and summer NDWI (Av.NDWI_4–7_). [70] also report an association between NWDI and WNV risk in Europe, specifically a positive association with anomalies in early June. NDWI is a proxy for the amount of water in the ecosystem, and low NDWI may be indicative of drought. Recent research suggests that drought may lead to subsequent localised increases in mosquito numbers and disease outbreaks [56,63]. Drought conditions may encourage the aggregation of both hosts (birds) and vectors (mosquitoes) at remaining water bodies, increasing rates of transmission [80], while potentially also influencing their vector competence [25]. *Culex pipiens* thrives during drought conditions by exploiting larval habitats with high organic matter (a consequence of drying content) and artificial containers not reliant on precipitation [81]. Furthermore, drought conditions may force amplifier bird species into urban and suburban areas where water is more freely available, thereby bringing competent hosts into contact with competent urban vectors [8]. Associations between dry summers and increased WNV outbreaks have been indicated in a number of US studies [82].

Wetlands [61,83,84], agricultural area [68,86], and urban infrastructure [66] have previously been reported to influence vector populations or WNV transmission [85]. In the current study, the detailed resolution of land use classes allows a distinction to be made between irrigated, and rain-fed crop lands. Irrigated croplands were positively associated with WNF, in accordance with research in the US where an increased human incidence of WNF was found to be associated with crop land cover and water catchment depressions in the otherwise semi-arid environment of Texas [87]. Rain-fed crop lands in the current study show no significant association with WNF, possibly because the more variable water supply is less favourable to mosquito populations.

A positive association with urban infrastructure has been repeatedly demonstrated in the US (e.g. [73,88,89], although with some exceptions and east-west differences e.g. [66]. This association is not apparent in the current study, and has not been reported in other European studies, although the largest outbreaks in the Old World, as in the New, have occurred in urban areas (e.g. Bucharest in 1995, Volgograd in 1999; Belgrade 2012; [90–92]). This probably reflects significant differences in urban planning and infrastructures design, demonstrating the importance of region specific analyses. Indeed, US suburban areas are often characterized by assemblages of houses surrounded by vegetation (gardens, public parks, trees) which provide the optimal habitat for interaction of synantropic birds, mosquitoes and humans. On the other hand, Old World cities, especially those with a longer history, typically include far less green space. Instead, small buildings surrounded by vegetation are more common in rural areas of the Old World, where WNF cases are common.

In the current study, results indicate a clear positive association of WNF cases with populated forests. ‘Populated forests’ are defined as those with relatively low human population density (1–10 individuals/km^2^), and are characterised by a mixture of forest, human habitation, farmlands and transitional habitat, and usually occur in agricultural areas [39]. In such areas houses are usually scattered within forest patches which provide refuge, nesting and feeding opportunity for birds, including species which are considered highly competent reservoir hosts for WNV [8]. The occurrence of natural and protected areas was not significantly associated with WNF, but it is likely that increased sprawl of urban settlements causes fragmentation of previously pristine forest systems, increasing contact rates between vectors (mosquitoes), competent reservoir hosts (birds) and dead-end hosts such as humans [64,85], therefore enhancing transmission from the sylvatic cycle [69].

Landscape structure, patchiness and matrix organization have previously been associated with variation in vector population and virus transmission at a local scale [85,86], but although our model suggests a negative association with an index of heterogeneity (the Pielou Index), the model support for this term is low and the result therefore inconclusive.

Increases in the risk of WNF emergence in humans may arise due to temporal extension of the transmission season, increased spatial extent of habitat suitable for hosts and vectors, or increased intensity of virus amplification and circulation among birds and mosquitoes. All of these variables are likely to vary with changes in climate, human population expansion and land use change. A better understanding of the environmental drivers of WNF may ultimately be used to map the spatial variation in risk across the continent. In conjunction with long range meteorological forecasts, environmental data might be used further, to forecast inter annual change. Together, these could be used to target public health actions and so mitigate risk from WNF emergence. At the current time however, a number of weaknesses in the data available mean that predictive modelling is unlikely to be accurate. In particular, the complexity of the viral transmission cycle remains poorly understood in the Old World, while human WNF incidence data are limited by geographic variation in the accuracy of diagnosis, the establishment of surveillance, and the organization of national reporting systems.

Further research is needed, integrating interdisciplinary research across human, veterinary and environmental health (i.e., in line with the ‘One Health’ initiative [93]). In particular, enhanced monitoring of WNV circulation in the environment (via the combined use of sentinel birds and the virological screening of mosquitoes) and the recording of environmental variables [94], and a European shared database collecting geo-localized reports of human WNF, would provide important advances in data quality. This combined with collection of socio-economic data, and enhanced environmental data (for example, inclusion of species specific bird density data or routes of migrations) would help the development of more reliable predictive models.

In conclusion, although further interdisciplinary research is required to develop accurate predictive models of WNF risk, the current study, making use of a multi model inference framework (for a complete overview on the topic please refer to the milestone paper on model selection by [48]), provides a valuable starting point, and successfully identifies and confirms a number of variables which are associated with WNV emergence in Europe, Asia and North Africa.
